# Independent Concentration Manipulation Using Sidewall-Driven Micromixer

**DOI:** 10.3390/mi15070869

**Published:** 2024-06-30

**Authors:** Toshio Takayama, Hayato Maki

**Affiliations:** Department of Mechanical Engineering, Tokyo Institute of Technology, 2-12-1 Ookayama, Meguro, Tokyo 152-8552, Japan

**Keywords:** micromixer, fluid vibration, soft actuator, drug discovery

## Abstract

Lab-on-a-chip technology has been developed to streamline biochemical experiments by providing experimental environments in microscopic areas. Due to the difficulty of mixing chemicals in such small channels, various micromixers have been created. Our proposed sidewall-driven micromixer offers easy fabrication and precise control over mixing concentrations. In our previous study, we successfully generated concentration gradients by simultaneously mixing multiple chambers using a single actuator. However, it is not possible to mix different chemicals in each chamber. In this study, we developed a sidewall-driven micromixer that enables independent mixing in each chamber by installing one actuator per chamber. Experimental results showed that different conditions were achieved in each chamber using both microbead-mixture water and colored water. Thus, this mixer can be used to manipulate concentrations regardless of whether the mixing targets are particles or fluids.

## 1. Introduction

In recent years, lab-on-a-chip technology has gained significant interest for biochemical experiments. A lab-on-a-chip provides an experimental environment of several tens of square millimeters, with primary functions including mixing, reaction, separation, and detection. Conventional methods perform these operations using containers larger than the scale of cells and drugs. Consequently, lab-on-a-chip technology is expected to improve experimental efficiency and reduce fluid waste [1,2,3].

The flow path inside a lab-on-a-chip is short, with a width of several tens to 100 μm, and has laminar flow, with a Reynolds number of approximately 1–10 [4,5,6]. Therefore, chemical mixing is time-consuming and problematic. This is crucial for biochemical experiments, including cell culture and drug reactions. Various micromixers have been proposed to address this issue and are classified into passive and active types based on their mixing mechanisms [7,8,9].

Passive mixers perform mixing by applying a flow rate via a pump along a flow path with a complex structure. Channel structures include those with splitting and recombination (SAR) [10,11,12,13], corners and curves [14,15,16], and injection nozzles [17,18,19]. Juraeva et al. developed a mixer that combined structures with corners and curves [20]. At the corners and curves, the flow velocities differed from those inside and outside the channel; therefore, the inertial forces varied. This generated vortices due to convection, which promoted mixing. In addition, when a high flow velocity flows around a sharp bend, horizontal vortices are created owing to flow separation. Using this combined structure, the mixer achieved mixing at a Reynolds number of approximately 10. Shah et al. developed a micromixer by combining SAR and curved structures [21]. When fluids with different flow velocities merge, turbulent flow forms due to chaotic convection. In the SAR structure, mixing is achieved through repeated mergers. Because mixing performance varies with the merging angle, Shah et al. optimized this angle via simulations and verified it experimentally. Mash et al. proposed a flow path using jet flow [22]. The jet flow is generated by widening a narrow channel, creating a vortex where the path expands, which stirs the fluid. Passive micromixers are advantageous due to easy fabrication and integration with different channels. However, they involve pressure loss, requiring a strong pumping function even for low Reynolds number flows. Additionally, most passive mixers aim for complete mixing, making it difficult to control mixing by concentration. Although passive mixers with three-dimensional structures offer high mixing performance, they are more challenging to create.

An active mixer mixes the fluids by applying energy using an actuator. The types of energy sources include acoustic vibrations [23,24,25], electricity [26,27], magnetism [28,29], and heat [30,31]. Lu et al. developed a mixer that performs mixing by applying acoustic vibrations to a flow path using a piezoelectric actuator [32]. The application of acoustic vibrations generates vortices at the corners and boundaries of the flow channel. Their mixer achieved high mixing performance through a combination of the channel shape and acoustic vibrations. However, because this method uses a flow path structure similar to that of an active mixer, a pressure loss occurs. Therefore, a pump is required in addition to the piezoelectric actuator. In addition, Kandalkar et al. developed a mixer by vibrating a conductor in a flow channel using the Lorentz force [33]. Magnets and conductive wires were incorporated into chips using template-assisted soft lithography. However, mixing is time consuming because the amplitude of the conductor is limited by the channel diameter. Chang et al. fabricated a mixer by applying a transverse flow to the main stream using a magnetic force [34]. However, this mixer requires time for mixing because of the addition of magnetic particles to the fluid. In addition, the effects of additives on biochemical experiments have been investigated. Matsushita developed a mixer that performs mixing via laser heating [35]. When bubbles are generated in the flow path by the laser, the temperature gradient causes Marangoni convection. This method achieved local mixing control. However, it is necessary to prepare special equipment such as lasers. Active mixers can control mixing, but embedding the actuator within the chip is time-consuming and difficult to integrate into different flow channels. Additionally, contamination by additives and the ecological compatibility of the mixing structure must be considered. Many mixers only perform mixing and require separate flow paths to trap cells and drugs [36,37,38]. Tanyeri et al. developed a channel that traps cells by generating vortices via colliding flows from two directions [39]. Such channels require a mechanism to generate the flow velocity. Hunt et al. developed a channel that traps cells and fluids using electrical induction [40]. This design requires incorporating an actuator into the chip. In both cases, a method is needed to connect the mixer and trap flow paths.

**Figure 1:**
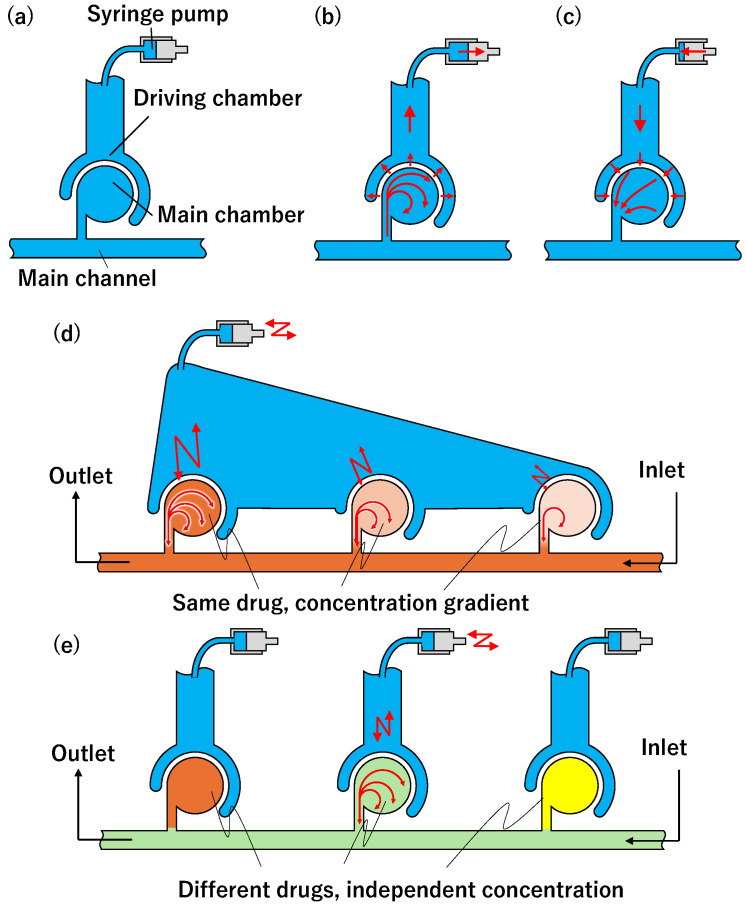
Channel structure of sidewall-driven micromixer. (**a**) Schematic of main channel, main chamber, and driving chamber. (**b**) Flow path when pulling syringe pump. (**c**) Flow path when pushing syringe pump. (**d**) Generation of concentration gradients by single actuator (our previous work). (**e**) Generation of independent concentrations by multiple actuators (proposition in this article).

Our previously developed sidewall-driven micromixer is easy to fabricate due to its single-layer structure, with the actuator located outside the chip. This design allows for direct mixing inside the chamber, enabling simultaneous trapping of cells and drugs. Additionally, mixing density can be controlled by adjusting the mixing time. The mixer comprises a main channel, main chamber, and driving chamber (Figure 1a). The main chamber branches off from the main channel, drawing in and mixing the fluid. The driving chamber is a flow path surrounding the main chamber and is connected to an external syringe pump. When pressure is applied via the syringe pump, the sidewall of the main chamber deforms, creating an internal flow. Because the internal flow pattern is asymmetric when the pump is periodically pushed (Figure 1b) and pulled (Figure 1c), a swirling flow is created and the fluid is mixed. This method is, as such, a soft actuator. Inside the proposed mixer, pressure force and strong swirling flow are applied. However, when real cells were introduced into the mixer, we confirmed that the cells survived [41]. Therefore, it has the possibility of being used for experiments such as cell reaction experiments with solutions with different densities. Koike et al. [42] developed mixers in which parallel chambers were driven by a single actuator, creating a concentration gradient in parallel chambers with one actuation (Figure 1d). However, this structure caused simultaneous mixing in all chambers, making it impossible to mix different solutions in each chamber. To drive each chamber independently, a separate driving chamber should be installed, as shown in Figure 1e. However, individual chamber actuation led to vibrations being transmitted to non-activated chambers, causing unintended mixing. This study aims to develop a mixer that can drive multiple chambers independently by eliminating vibration transmission between chambers and can enable the generation of different concentration states in different chambers.

## 2. Microchannel Design and Fabrication Method

### 2.1. Channel Design

#### 2.1.1. Preliminary Experiment to Identify the Cause of Mixing Interference

**Figure 2:**
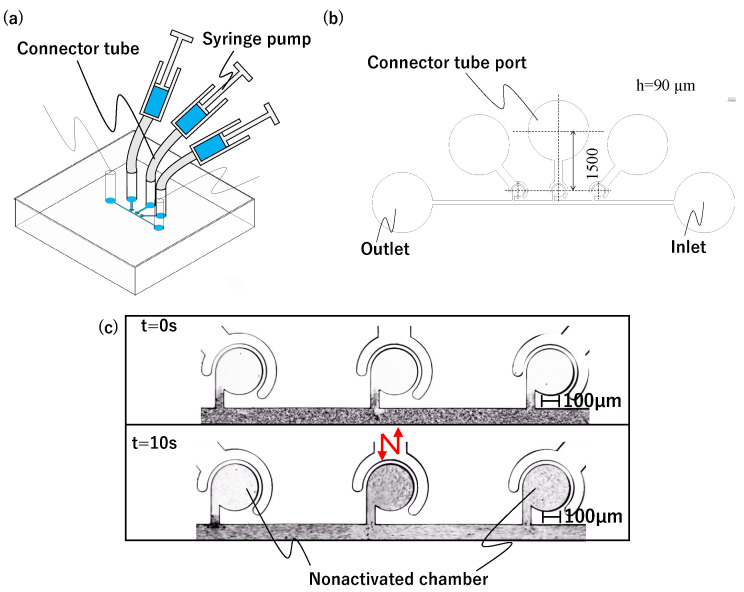
Mixer with an actuator connected to each chamber. (**a**) Schematic of connection of chip, syringe pumps, and connector tubes. (**b**) Channel design of the prototype. (**c**) Mixing results in the prototype.

**Figure 3:**
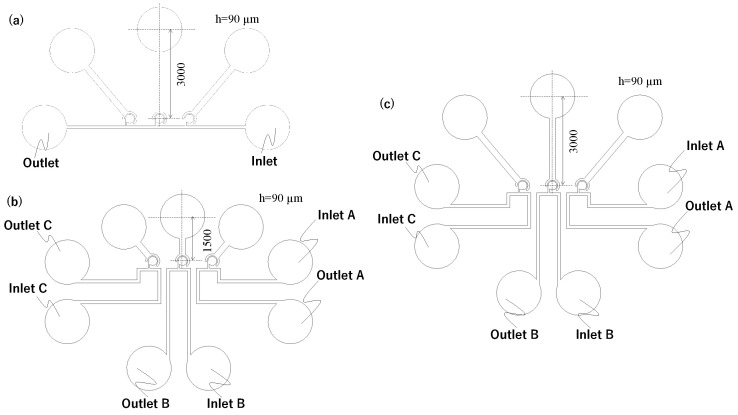
Verification flow path for interference drive, where (**a**–**c**) correspond to Table 1.

**Figure 4:**
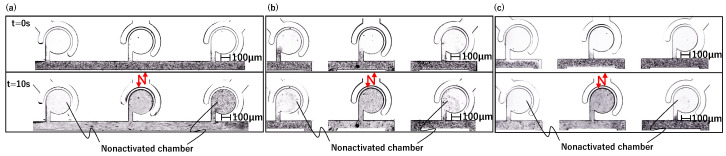
Mixing in the verification flow path, where (**a**–**c**) correspond to Table 1.

The sidewall-driven micromixer has an issue whereby non-activated chambers can be interfered with and start to mix when another chamber is activated. Figure 2a,b shows a preliminary prototype flow path with separate driving chambers for each main chamber. Figure 2a is a schematic diagram of the entire chip. By injecting water from the inlet, the outlet, and all connector tube ports, the air in the chamber is discharged through PDMS by its air permeability, and channels and chambers can be filled with water. Each of the three driving chambers was connected to a single-syringe pump, allowing independent operation. Figure 2b shows the flow path design. The driving chamber length is 1.5 mm, and all three main chambers are connected to a straight main channel. Other design details are the same as those described in Section 2.1.2. First, we considered that the mixers could be independently driven using this channel design. However, during experiments wherein we injected 1 μm microbead-mixed water into the main channel and operated the central mixer at 1000 Hz using a piezo actuator to push and pull the syringe pump, mixing was observed in the non-activated left and right chambers (Figure 2c). We repeated these experiments multiple times with various channel designs and identified two potential causes of interference. One is vibrations transmitted via the liquid in the main channel, and the other is vibrations transmitted via the elastic PDMS material itself. To investigate the effects of this interference through the main channel and the length of the driving chamber, we created three types of flow channels with different shapes for the driving chamber and main channel, as shown in Figure 3. The differences in Figure 3a–c are summarized in Table 1. Compared to the prototype flow path shown in Figure 3a, the length of the driving chamber in Figure 3b is doubled, and the main channel is separate for each chamber. Figure 3c incorporates both modifications. Figure 4 shows photographs of mixing in these three types of channels when the central mixer is driven at 1000 Hz. The channels in Figure 4a–c also correspond to Table 1.

As shown in Figure 4a,b, mixing occurs in a non-activated chamber, but in Figure 4c, no mixing occurs. Thus, the cause of interference drive can be considered as follows:

1. The main channel is separated in order to prevent the transmission of vibration, resulting in the chip vibration around the connector tube port being possibly transmitted to the non-activated chambers due to the elasticity of PDMS (Figure 4b).
2. The interfering drives can be reduced by lengthening the driving chambers and buffering the fluid vibration in the main channel by separating the main channels. Therefore, by lengthening the driving chambers, the vibration transmission through PDMS can be suppressed (Figure 4c).
3. The vibration transmitted through PDMS was suppressed by lengthening the driving chambers. However, the repeated suction and discharge of liquid by the activated mixer generate vibrations in the main channel. These vibrations are transmitted to the non-activated chambers via the main channel, resulting in mixing (Figure 4a).

#### 2.1.2. Channel Design to Avoid Vibration Interference

**Figure 5:**
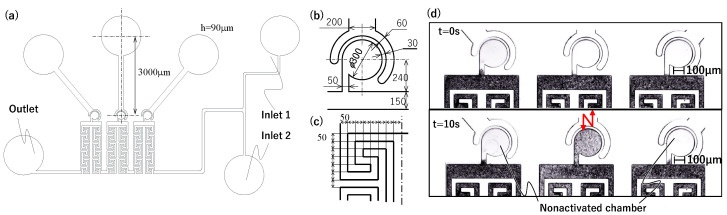
Flow paths that can be driven independently. (**a**) Channel design. (**b**) Detail of the mixer. (**c**) Detail of the corners of the main channel. (**d**) Mixing when the center mixer is activated.

Based on the above considerations, we designed a flow path to reduce vibration interference. Figure 5a shows the overall layout of all channels. The details are also shown in Figure 5b,c. The driving chamber is 3000 μm long, and the main channel is long, narrow, and has many corners. The fluid resistance at the constrictions and vortex turbulence at the corners of the main channel help reduce the transmission of fluid vibrations. Although a longer main channel can further reduce interference, it also poses challenges such as an increased likelihood of clogging with drugs and cells and longer fabrication time. Considering the irradiation range of the exposure device, we created a channel with 76 corners between the chambers and a channel width of 50 μm. Additionally, two inlets were provided to allow the injection of multiple fluids. Using this flow path, mixing was performed in the central chamber, similar to the prototype. Figure 5d shows a photograph from a preliminary experiment to confirm the effect of the cornered main channel. Almost no mixing occurred in the non-activated chambers, which suggests that independent driving is possible. Based on this, we conducted an experiment using this mixer to generate different concentration states in different chambers.

### 2.2. Microchip Fabrication Method

**Figure 6:**
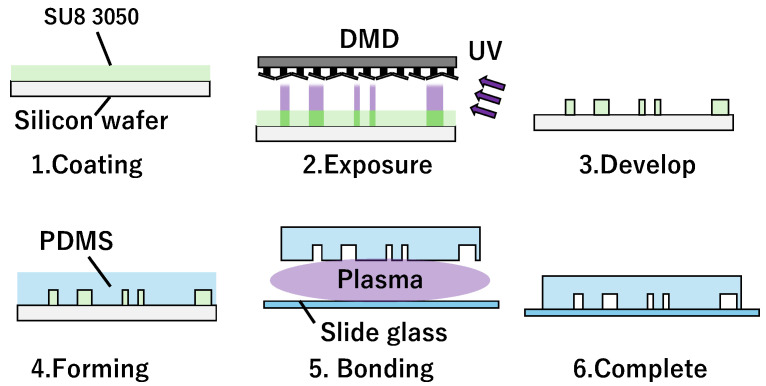
Photolithography steps, where 1 to 6 are procedures of coating, exposure, develop, forming, bonding, and completing to make the chip.

The main channel was created using photolithography. The Si wafer was coated with SU8-3050 (KAYAKU Advanced Materials, West Borough, MA, USA) to a thickness of 90 μm (Figure 6-1). The channel structure was exposed using a mask-less exposure device (PALET; NEOARK Corporation, Tokyo, Japan) (Figure 6-2). It was then developed using a thinner, forming a mold (Figure 6-3). Polydimethylsiloxane (PDMS) (SILPOT184; Dow, Midland, MI, USA; main agent:hardener = 10:1 (mass ratio)) was poured into the mold to form a flow path (Figure 6-4). Finally, the hardened PDMS and glass slides were bonded via plasma treatment (Figure 6-5) to fabricate the microchip (Figure 6-6).

### 2.3. Experimental Setup

**Figure 7:**
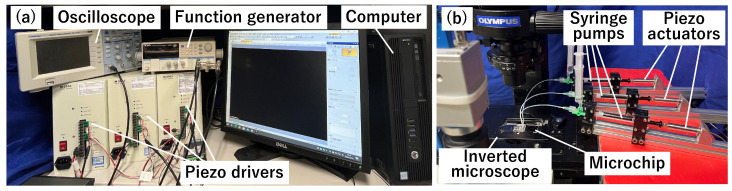
Experimental device. (**a**) Control system of the actuators. (**b**) Actuators and the microchip.

Figure 7a shows the actuator control equipment. As shown, piezo-actuators (Pst150/5/40; Shoei System, Tokyo, Japan) were used to drive the syringe pumps (2.5MDF-LL-GT; Trajan Scientific Australia Pty Ltd., Ringwood, Australia). Each of the three piezo-actuators was connected to an independent piezo-driver (M-2503; MESS-TEK, Saitama, Japan). A function generator (FG-274, Texio Technology Corporation, Aichi, Japan) output a 1000 Hz, 3 V square wave, which was amplified 15 times using piezo-drivers. A voltage was supplied to each piezo-actuator to push and pull the liquid periodically. The input voltage was measured using an oscilloscope (DS-5110B; Iwtsu Electric Co. Ltd., Tokyo, Japan). A computer was used to switch between the three piezo-drivers. Each piezo-driver was connected to a digital input/output device (DO-16TY-USB, Contec Co., Osaka, Japan). Furthermore, the computer and device were connected via a USB connection, enabling the piezo-actuators to be turned on and off using a computer. Figure 7b shows the piezo-actuator and microchip setup. Each piezo-actuator and microchip were connected via a polytetrafluoroethylene tube. The state of the microchip was observed using an inverted microscope (IX73P1F; Olympus, Tokyo, Japan), and the video was imported into the computer at a frame rate of 30 fps.

### 2.4. Experimental Procedure

#### 2.4.1. Stirring Experiments Using Microbeads of Different Sizes

**Figure 8:**
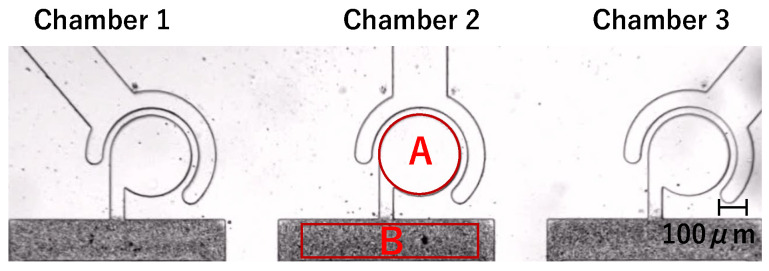
Experimental device. (A) Control system of the actuators. (B) Actuators and the microchip.

Stirring experiments were performed using microbeads with diameters of 1 μm and 5 μm. Figure 8 shows the schematics of Chambers 1, 2, and 3. The microbeads were stirred according to the following procedure:

1. The main channel, all main chambers, and all driving chambers were filled with pure water. Pure water and 1 μm microbead-mixed solution were connected to Inlets 1 and 2, respectively.
2. A 1 μm microbead-mixed solution was injected from Inlet 2 to fill the main channel. The injection task was done manually with a syringe.
3. Before mixing, the flow in the main channel was halted, and the mixer was driven in Chambers 1 and 2 to stir the fluid in each chamber.
4. Pure water was injected from Inlet 1 to flush out the beads from the main channel. The solution at Inlet 2 was replaced with a 5 μm microbead-mixed solution.
5. A 5 μm microbead-mixed solution was injected from Inlet 2 to fill the main channel. This was also done manually.
6. Before mixing, the flow in the main channel was halted, and the mixer was driven into Chambers 2 and 3 to stir the fluid inside each chamber.

#### 2.4.2. Stirring Experiments Using Differently Colored Waters

Because the natural diffusion of fluids is greater than that of solid particles such as microbeads, unintended inflows can occur even in non-activated chambers. We conducted stirring experiments using red, blue, and yellow water prepared with food coloring using the following procedure to investigate whether stirring was independent.

1. The main channel, main chambers, and driving chambers were filled with pure water, which was connected to Inlet 1, and red water was connected to Inlet 2.
2. Red water was injected from Inlet 2 to fill the main channel.
3. The mixer in Chamber 1 was driven to stir the fluid inside it.
4. Pure water was injected from Inlet 1 to expel colored water from the main channel and clean the flow path. Inlet 2 was then replaced with water.
5. Steps 2 and 3 were repeated except for in Chamber 2, which was stirred. Furthermore, in Step 4, Inlet 2 was replaced with yellow water.
6. Steps 2 and 3 were repeated except for in Chamber 3, which was stirred.

### 2.5. Evaluation Method

The concentration was evaluated using MATLAB software. In each experiment, the mixture ratio was defined as the mixing index, which was calculated from the start to the end of the stirring. However, a time lag of approximately 1–2 s existed between the power switching of each piezo driver and the response of each piezo actuator. Therefore, the start and end times for stirring were set as the times at which the fluid to be stirred began to flow and the fluid vibration stopped in the main chamber, respectively. In the experiments using microbeads of different sizes, the brightness inside each chamber was examined experimentally using microbeads of different sizes. In the *i*th frame of a video shot in grayscale, $A_{i}$ represents the brightness of Area *A*, as shown in Figure 8. In addition, the brightnesses of Areas A and B before stirring are denoted as $A_{0}$ and $B_{0}$, respectively, and the mixture ratio $N_{i}$ of the *i*th frame is defined as

(1) $$N_{i}=\frac{A_{i}-A_{0}}{B_{0}-A_{0}}.$$

The mixture ratio $N_{i}$ was calculated for Chambers 1, 2, and 3 until the end of stirring, and changes in the mixture ratios over time were investigated. In the stirring experiments using differently colored water, we investigated the amounts of red, blue, and yellow colors in each chamber using the following procedure:

1. An image of the red water in the flow channel was captured on the RGB scale and divided into R, G, and B images. By examining the brightness, we determined the R, G, and B values of the red water as $R_{R}$, $R_{G}$, and $R_{B}$, respectively. Similarly, those of the blue water, $B_{R}$, $B_{G}$, and $B_{B}$, and those of the yellow water, $Y_{R}$, $Y_{G}$, and $Y_{B}$, were determined.
2. A video was captured in the RGB scale, and the first frame was divided into R, G, and B images. For each image, the brightness of Area A was calculated as $R_{0}$, $G_{0}$, $B_{0}$. Using these values, the following matrix was obtained:
  (2) $$P=\left[\begin{array}{ccc} R_{R}-R_{0} & B_{R}-R_{0} & Y_{R}-R_{0}\\ R_{G}-G_{0} & B_{G}-G_{0} & Y_{G}-G_{0}\\ R_{B}-B_{0} & B_{B}-B_{0} & Y_{B}-B_{0} \end{array}\right]$$
3. The *i*th frame of the captured video was divided into R, G, and B images. For each image, the brightness of area A is calculated using $R_{i}$, $G_{i}$, $B_{i}$.
4. The mixture ratios of the red, blue, and yellow waters in the chamber, $N_{R_{i}}$, $N_{B_{i}}$, and $N_{Y_{i}}$, respectively, were defined and calculated as follows:
  (3) $$\left[\begin{array}{c} N_{R_{i}}\\ N_{B_{i}}\\ N_{Y_{i}} \end{array}\right]=P^{-1}\left[\begin{array}{c} R_{i}-R_{0}\\ G_{i}-G_{0}\\ B_{i}-B_{0} \end{array}\right]$$
5. Three mixture ratios were obtained for each chamber in the *i*th frame. Changes in the mixture ratios in each chamber over time until the end of stirring were investigated.

## 3. Results

### 3.1. Stirring Experiments Using Microbeads of Different Sizes

**Figure 9:**
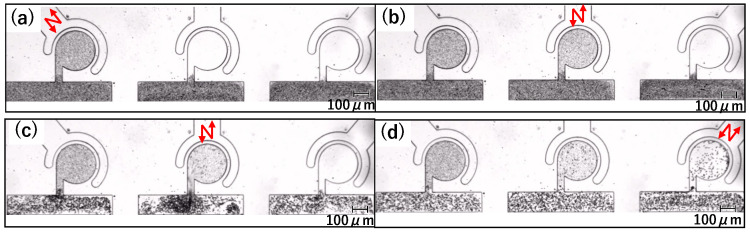
Photographs of experiments using mixing beads of different sizes. (**a**) 1 μm beads are mixed in Chamber 1. (**b**) 1 μm beads are mixed in Chamber 2. (**c**) 5 μm beads are mixed in Chamber 2. (**d**) 5 μm beads are mixed in Chamber 3.

**Figure 10:**
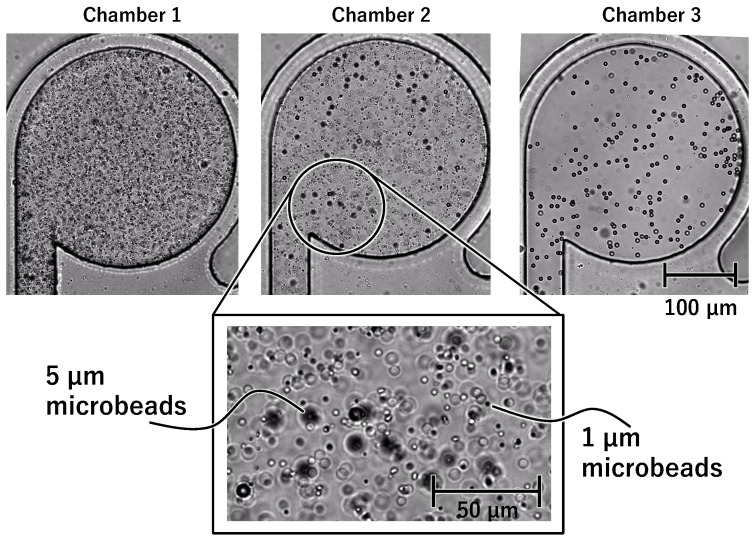
Photos of all three chambers after mixing.

**Figure 11:**
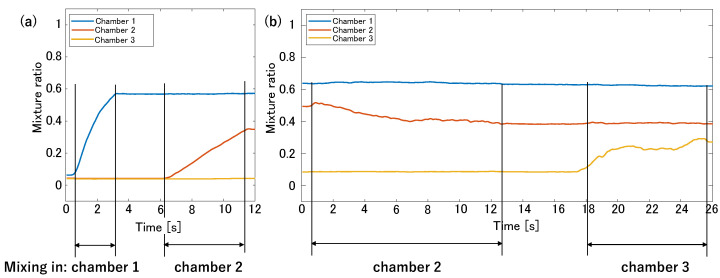
Mixing ratios of the microbead-mixed solution. The vertical axis represents the mixing ratio, and the horizontal axis represents time [s]. (**a**) 1 μm beads are mixed sequentially in Chambers 1 and 2. (**b**) 5 μm beads are mixed sequentially in Chambers 2 and 3.

Figure 9 shows photographs of the three main chambers during stirring. The photographs in Figure 9a–d are taken immediately after stirring the 1 μm beads in Chamber 1, the 1 μm beads in Chamber 2, the 5 μm beads in Chamber 2, and the 5 μm beads in Chamber 3, respectively. During each stirring, very few beads entered the non-activated chamber. Figure 10 shows the photographs of the three main chambers after a series of stirring steps. Chambers 1–3 contain the 1 μm, 1 μm and 5 μm, and 5 μm beads, respectively. An enlarged view of the circled area from Chamber 2 is shown. This confirms that Chamber 2 contained both types of microbeads. Figure 11 shows plots of the mixture ratios under each stirring condition. The vertical axis represents the mixture ratio, and the horizontal axis represents time [s]. Figure 11a corresponds to the case when the 1 μm beads are stirred sequentially in Chambers 1 and 2. Figure 11b corresponds to the case when the 5 μm beads are stirred sequentially in Chambers 2 and 3. At each stirring time, the mixture ratio in the non-activated chamber hardly changes. The mixture ratio of Chamber 2 at t = 0 in Figure 11b is higher than at t = 12 in Figure 11a. This was because the brightness of Area A in Figure 8 changed as the 1 μm beads in the main channel were replaced with the 5 μm beads, and the concentration of the 5 μm microbead-mixed solution was lower than that of the 1 μm microbead-mixed solution. In addition, Figure 11b shows that the concentration in Chamber 2 decreases during stirring, probably because Chamber 2 was already filled with beads before driving. Therefore, the brightness inside the chamber was lower than that in the main channel. These results confirm that beads of different sizes were stirred in different chambers.

### 3.2. Stirring Experiments Using Differently Colored Waters

**Figure 12:**
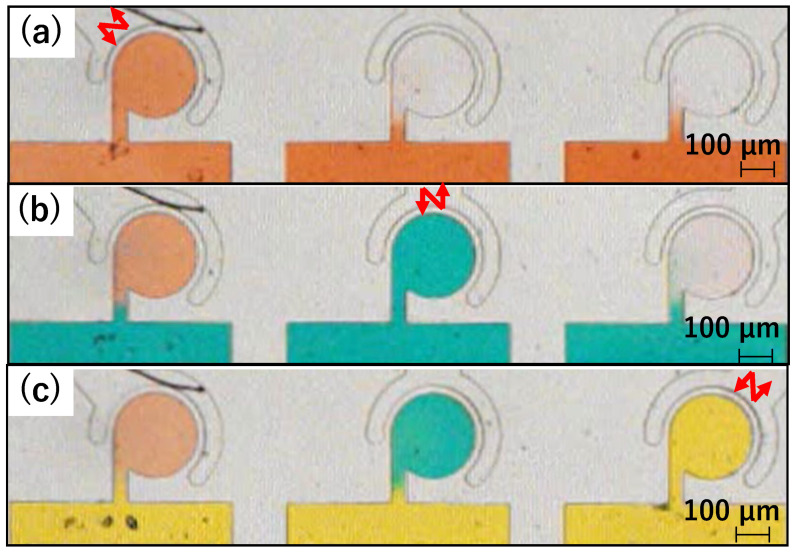
Photographs of stirring experiments using colored water. (**a**) Red water is mixed in Chamber 1. (**b**) Blue water is mixed in Chamber 2. (**c**) Yellow water is mixed in Chamber 3.

**Figure 13:**
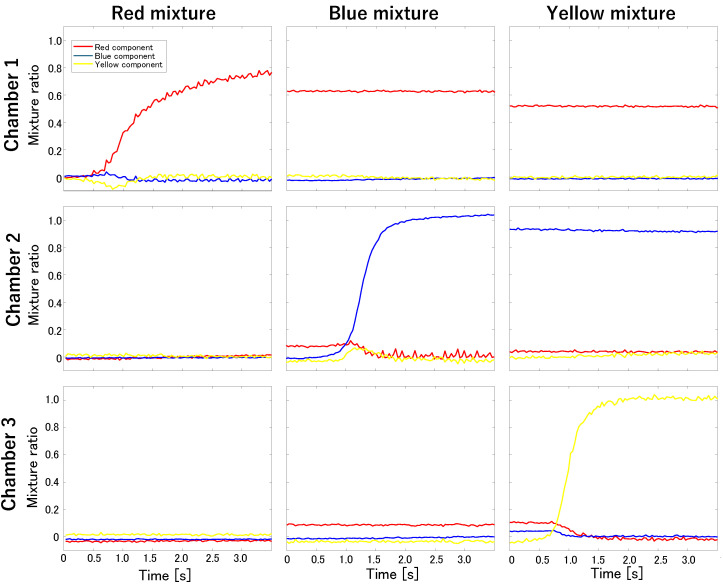
Mixing ratio of the colored water. The vertical axis represents the mixing ratio, and the horizontal axis represents time [s]. The red, blue, and yellow lines represent the mixing ratios of their respectively colored water in the chamber.

Figure 12 shows photographs of the main chambers during stirring. The photographs in Figure 12a–c were taken immediately after stirring the red water in Chamber 1, blue water in Chamber 2, and yellow water in Chamber 3, respectively. In all three chambers, after the colored water filled the main channel, it flowed near the chamber entrance, but no further flow occurred, and it stopped in the narrow channel. Additionally, the colored water was added to each chamber after stirring. Throughout the experiment, although the concentration of colored water was diluted, no unintended colored water flowed into the non-activated chambers. Figure 13 plots the mixture ratios for each stirring condition. The vertical axis represents the mixture ratio, and the horizontal axis represents time [s]. The red, blue, and yellow lines represent the color mixture ratios of the corresponding colored water inside each chamber. The change in the mixture ratio in the non-activated chamber was minimal under each stirring condition. These results indicate independent stirring of differently colored water in the three chambers.

## 4. Discussion

### 4.1. Microbead Size and Stirring Time

Stirring experiments using beads of different sizes revealed that the stirring time depends on the bead size. Figure 13 shows that in Chamber 1, where only 1 μm beads were stirred, the mixture ratio reached 0.8 in approximately 2 s. However, in Chamber 3, where only 5 μm beads were stirred, the mixture ratio was less than 0.3 even after approximately 7 s. The varying mixing abilities in each chamber are discussed further in the subsequent subsection. However, the difference in mixing speed was excessively large. This indicates that as the mass of the beads increased, more time was required for thorough stirring. When stirring objects with large masses, widening the chamber entrance or increasing the height of the flow path may be necessary in order to reduce the flow resistance. However, if the objective is to mix particles and solutions in the chamber using such a mixer, the solutions may unintentionally flow into the chambers due to natural diffusion. Balancing between reducing stirring time and managing natural diffusion effects is crucial during the design process. The acceptability of natural diffusion varies depending on the intended application, influencing the design balance accordingly. Determining design methods that account for acceptable levels of natural diffusion will be addressed in our future work.

### 4.2. Actuator and Mixture Ratio

Figure 11a demonstrates that for the mixing of identical microbeads, the stirring speeds in Chambers 1 and 2 differ, leading to distinct mixing ratios. Additionally, as depicted in Figure 11, the stirring times for the red and blue/yellow water differ. Exchanging the actuators for Chambers 1 and 2 resulted in a change in the mixing performance of the chambers, indicating that even a slight difference in actuator performance affects the stirring speed and mixing ratio. While adjusting the stirring time can produce a uniform concentration, low actuator performance may lead to natural diffusion, causing flow into the non-activated chamber. Conversely, excessively high actuator performance may cause inflow due to acoustic vibrations. Therefore, investigating the relationship between the actuator performance and mixing ratio for independent driving and concentration control is necessary. Furthermore, to equalize varying mixing performances among actuators, real-time monitoring of the main chamber and feedback control should be implemented. Moreover, although a sidewall-driven micromixer facilitates fluid and particle flow into chambers, diluting the concentration necessitates injecting a diluting fluid into the main channel, which is time-consuming. Hence, future studies require the development of a suitable control system.

### 4.3. Inflow during Fluid Exchange

Stirring experiments using differently colored water revealed inflow occurrences in the chambers during fluid exchange. As depicted in Figure 13, when red water is stirred in Chamber 1, no stirring is observed in Chambers 2 and 3. However, prior to stirring blue water in Chamber 2, both Chambers 2 and 3 contained red water. This is attributed to the pressure applied to the main channel during fluid exchange, which caused inflow into these chambers due to the sidewall’s susceptibility to deformation by the pressure from the main channel. Additionally, the mixture ratio of the chamber that was stirred decreased due to the flow of pure water during cleaning. To address this issue, minimizing pressure application during fluid exchange is necessary. However, even if fluid exchange is time-consuming, natural diffusion may cause inflow into the chambers; hence, rapid fluid exchange while controlling the pressure inside the flow path is crucial. A future challenge lies in controlling the pressure and flow velocity along the main channel.

### 4.4. Discharge during Stirring

In Figure 13, plotting the mixture ratios obtained from stirring experiments using differently colored water reveals that the red water entering Chambers 2 and 3 during fluid exchange decreases during subsequent stirring. Furthermore, Figure 11, which is based on stirring experiments using beads of different sizes, indicates that the amount of 1 μm beads stirred in Chamber 2 decrease when the 5 μm beads were stirred afterward. Consequently, chemicals and substances contained within the chambers may be discharged during subsequent mixing. Thus, adjusting their concentrations in anticipation of discharge when mixing different chemicals or trapping cells becomes necessary. In future research, investigation of the relationship between mixing time, concentration, and emission and exploring mixing methods using feedback control will be pursued.

### 4.5. Demonstration to Mix Differently Colored Waters

**Figure 14:**
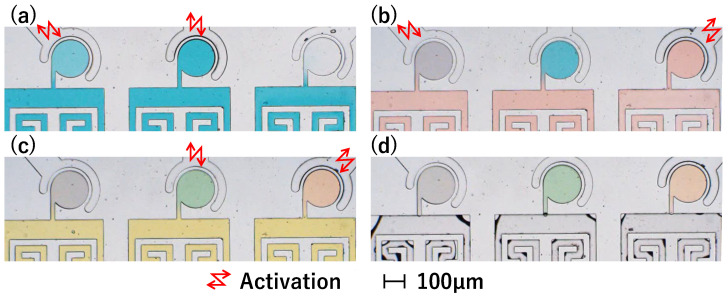
Demonstration to mix differently colored waters. (**a**) Injection of blue water and activation of Chambers 1 and 2. (**b**) Injection of red water and activation of Chambers 1 and 3. (**c**) Injection of yellow water and activation of Chambers 2 and 3. (**d**) Injection of air into the main channel.

We conducted a demonstration showing that this mixer can independently mix differently colored waters in each chamber. The procedures were as follows: (a) blue-colored water was drawn into Chambers 1 and 2, (b) red-colored water was drawn into Chambers 1 and 3, (c) yellow-colored water was drawn into Chambers 2 and 3, and (d) air was injected into the main channel to isolate all chambers. The driving time for each mixer was 3 s, and the injection time for the colored water into the main channel was approximately 3 s. The injection was done manually, so the time was not precise. As shown in Figure 14, the proposed device successfully mixed the colored waters, and the colors of the chambers became purple, green, and orange.

## 5. Conclusions

In our previous studies, sidewall-driven micromixers were used to mix multiple chambers simultaneously using a single actuator, making it impossible to mix different chemicals in each chamber. In this study, we developed a mixer with one actuator attached to each chamber, aiming to drive each chamber independently. In this mixer, unintended interference occurs in the non-activated chambers owing to the transmission of vibrations through the chip and fluid. By altering the shapes of the driving and main channels, each main chamber can be driven independently. We conducted experiments to verify the ability to mix particles and fluids using this mixer. For this experiment, we used microbeads and colored water, and independent stirring was performed in each chamber. This confirms that particles such as cells and fluids such as drugs can be mixed using a sidewall-driven micromixer. However, inflow occurred during fluid exchange and cleaning. Once the agitated fluid mixed with the other fluids, it was discharged. Furthermore, the mixing performance differed depending on the actuator used. Resolving these issues is necessary to create an arbitrary environment for each chamber. One solution is to improve the flow path design and experimental methods in order to minimize the fluid exchange between the main channel and the main chambers except when the mixer is driven. In addition, to reduce the difference in mixing caused by the actuator, real-time visual feedback control based on concentrations can be applied. Future prospects include simulations and experiments to optimize flow paths, pumps to control the flow rate and pressure to prevent inflow, and methods to monitor and control the concentration. In this study, each mixer was driven by a piezo-actuator, which generated a large thrust force capable of reliably moving any syringe pump, even during prolonged use. However, piezo-actuators are expensive and not conducive to widespread use. Therefore, in future work, we aim to explore driving the proposed independently driven micromixers using air pressure.

## Figures and Tables

**Table 1:** Characteristics of verification channel.

	Driving Chamber Length	Main Channel
(a)	3000 μm	Continuous
(b)	1500 μm	Separate
(c)	3000 μm	Separate

## Data Availability

The datasets used and/or analyzed during the current study are available from the corresponding author on reasonable request.
